# Immunogenicity of plant‐produced porcine circovirus‐like particles in mice

**DOI:** 10.1111/pbi.13097

**Published:** 2019-03-10

**Authors:** Cornelius J. Gunter, Guy L. Regnard, Edward P. Rybicki, Inga I. Hitzeroth

**Affiliations:** ^1^ Biopharming Research Unit Department of Molecular and Cell Biology University of Cape Town Cape Town South Africa; ^2^ Division of Medical Virology Department of Pathology and Institute of Infectious Diseases and Molecular Medicine Faculty of Health Sciences University of Cape Town Cape Town South Africa; ^3^ Institute of Infectious Disease and Molecular Medicine University of Cape Town Cape Town South Africa

**Keywords:** porcine circovirus type 2, plant‐produced, virus‐like particles, purification, vaccine

## Abstract

Porcine circovirus type 2 (PCV‐2) is the main causative agent associated with a group of diseases collectively known as porcine circovirus‐associated disease (PCAD). There is a significant economic strain on the global swine industry due to PCAD and the production of commercial PCV‐2 vaccines is expensive. Plant expression systems are increasingly regarded as a viable technology to produce recombinant proteins for use as pharmaceutical agents and vaccines. However, successful production and purification of PCV‐2 capsid protein (CP) from plants is an essential first step towards the goal of a plant‐produced PCV‐2 vaccine candidate. In this study, the PCV‐2 CP was transiently expressed in *Nicotiana benthamiana* plants via agroinfiltration and PCV‐2 CP was successfully purified using sucrose gradient ultracentrifugation. The CP self‐assembled into virus‐like particles (VLPs) resembling native virions and up to 6.5 mg of VLPs could be purified from 1 kg of leaf wet weight. Mice immunized with the plant‐produced PCV‐2 VLPs elicited specific antibody responses to PCV‐2 CP. This is the first report describing the expression of PCV‐2 CP in plants, the confirmation of its assembly into VLPs and the demonstration of their use to elicit a strong immune response in a mammalian model.

## Introduction

Porcine circovirus type 2 (PCV‐2) belongs to the genus *Circovirus* in the family *Circoviridae* and is one of the smallest autonomously replicating DNA viruses infecting mammals. The virus has a circular ssDNA genome of ~2 kb in size, encapsidated by a nonenveloped 17 nm diameter icosahedral capsid composed of a single coat protein (CP; Finsterbusch and Mankertz, 2009). PCV‐2 was first isolated in 1991 from Canadian piglets which presented with postweaning multisystemic wasting syndrome (Harding and Clark, 1997). This syndrome is the highest contributor to economic losses seen in the global swine industry and was the first disease associated with PCV‐2 (Segalés *et al*., 2013). However, it has since been identified as just one of several syndromes associated with PCV‐2. PCV is transmitted mainly via the oral‐nasal route, but any excretions and secretions can be responsible for viral transmission (Gillespie *et al*., 2009). Recently, reports have emerged of a circulating PCV‐3 strain currently known to be associated with dermatitis and nephropathy syndrome as well as reproductive failure in swine and its full characterization is still underway (Ku *et al*., 2017; Palinski *et al*., 2017).

Stringent PCV‐2 vaccination practices have seen an effective control of the virus and its associated diseases in swine herds globally (Afghah *et al*., 2016). There are currently five commercial vaccines against PCV‐2: two of these are whole‐killed virus vaccines and three are subunit‐based vaccines. All five PCV‐2 vaccines are based on the genome ORF2‐encoded virus CP as it contains the main determinants capable of inducing protective and lasting humoural immunity (Ren *et al*., 2016). It has been demonstrated by expression in insect cells that the CP is capable of self‐assembling into virus‐like particles (VLPs), which are morphologically indistinguishable from native PCV‐2 virions (Chae, 2012). The production and use of VLP‐based vaccines are advantageous compared to subunit protein(s), as they provide the efficacy of whole‐killed vaccines but do not have the safety concerns associated with production and inactivation of virulent live virus. VLPs made in insect‐, yeast‐ and bacterial cell‐based production systems are capable of eliciting humoural‐ and cell‐mediated immunity in experimental animal trials (Li *et al*., 2016; Nainys *et al*., 2014; Xi *et al*., 2016). Live attenuated vaccines elicit strong immune responses but carry a risk of reversion to virulence, whereas inactivated vaccines are safer to use if not to make, but are generally not as effective (Chambers *et al*., 2016). VLP‐based vaccines combine the advantages of both and have been the focus of many current vaccine production strategies. Although the recombinant human hepatitis B virus and the human papillomavirus VLP‐based candidates have seen commercial success, there are currently no veterinary VLP‐based vaccines on the market (Karuppannan and Opriessnig, 2017).

Recombinant protein production methods are traditionally based on microbial, insect or mammalian cell cultures, which rely on the use of expensive growth media and sterile bioreactors. The high demand for biomedically and industrially useful proteins requires simpler, safer and sustainable production systems that are scalable. Plant‐based transient expression systems fill these criteria, from laboratory scale expression through to industrial‐scale manufacturing of bioactive proteins. Plant cells are capable of performing essential posttranslational modifications to recombinant proteins and also carry negligible risk of cross‐contamination with animal pathogens, a concerning drawback with conventional eukaryotic expression systems (Fahad *et al*., 2015; Pogue and Holzberg, 2012; Rybicki, 2009). In addition, the upstream requirements for transient expression of proteins in plants are a BSL‐1 laboratory and a greenhouse to cultivate plants, making this technology accessible to a wider research community for exploration.

The downstream processing of recombinant proteins adds considerably to the raw biomass production cost and is a major focus for any competitively viable vaccine candidate. Near complete purification of correctly folded PCV‐2 CP and VLPs has been achieved using affinity tag columns followed by size‐exclusion chromatography (Wu *et al*., 2016; Zhang *et al*., 2016) or ion‐exchange chromatography (Xi *et al*., 2016; Zaveckas *et al*., 2015). These methods are successful at purifying the peptide of interest, but the addition of an affinity tag and need for chromatography columns add considerably to cost, as well as to the production complexity when removing the affinity tags during recombinant protein purification. PCV‐2 VLPs have however been successfully purified from other expression systems using density gradient centrifugation separation techniques (Li *et al*., 2016; Nainys *et al*., 2014; Wu *et al*., 2012).

The aim of this study was to examine the viability of expressing and purifying PCV‐2 CP using a whole‐plant transient expression system. Successfully purifying competitive amounts of PCV‐2 CP from plants with the ability to elicit and immune response in a mammalian model, forms part of the essential first step towards the goal of a plant‐produced PCV‐2 vaccine candidate. This was investigated via expression time trials on *N. benthamiana* plants infiltrated with recombinant *Agrobacterium tumefaciens* containing the CP‐encoding expression vector, the purification and quantitation of the PCV CP yield and the investigation of extracts by electron microscopy. Immunogenicity of the product was investigated by injection of mice, compared to a commercial subunit vaccine.

## Results

### Expression, purification and quantitation of plant‐produced PCV‐2 CP

**Figure 1:**
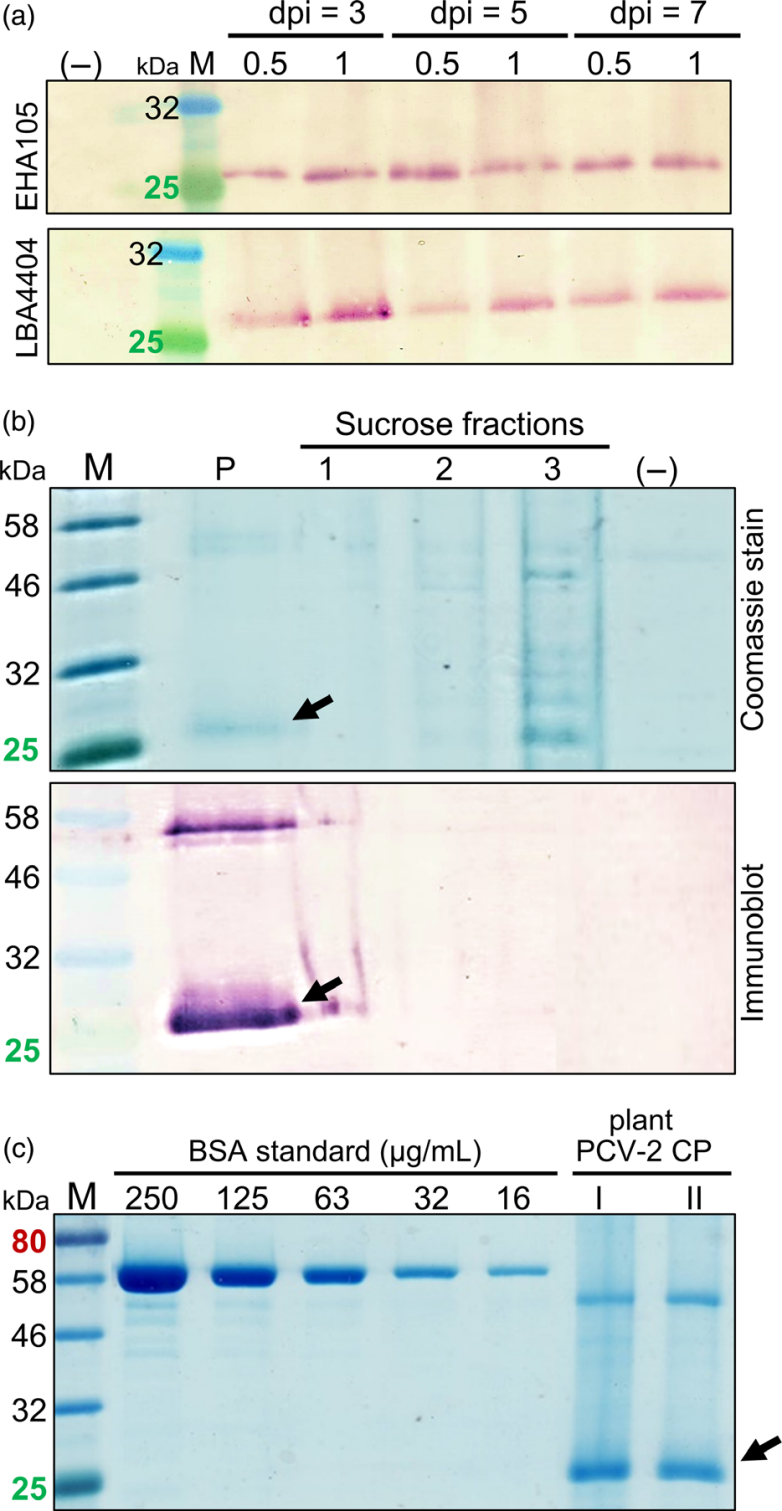
Expression, purification and quantification of plant‐produced PCV‐2 CP (27 kDa). (a) Immunoblots of plant‐made PCV‐2 CP probed with rabbit anti‐PCV2 CP antibody comparing harvest day post infiltration (dpi), *A. tumefaciens* strains EHA105 and LBA4404 and infiltration OD_600_ of 0.5 or 1.0. Lanes were loaded with equal volumes of plant homogenate for direct comparison. (b) Coomassie Blue‐stained SDS‐PAGE gel and corresponding immunoblot of plant‐produced PCV‐2 CP (black arrows) sucrose gradient fractions (1–3) and resuspended pellet (P). (c) Two independently expressed and partially purified recombinant 27 kDa PCV‐2 CP samples I and II resolved on Coomassie Blue‐stained SDS‐PAGE for densitometric analysis and quantification. Empty pEAQ‐HT vector control (–) and molecular weight marker (M) included. Lanes were loaded with equal volume of sample and standard.

Recombinant PCV‐2 CP protein production in plants was optimized based on comparison of *A. tumefaciens* strains, varying the optical density of infiltrates and determining the optimum harvest day post infiltration (dpi). There was no notable difference in expression between the two *Agrobacterium* strains. The LBA4404 strain had the highest expression when the OD_600_ infiltration was 1.0 from 3 dpi onwards, compared to EHA105 which had the highest expression 5 dpi and at an OD_600_ infiltration of 0.5 (Figure 1a). An OD_600_ infiltration of 0.25 resulted in very low protein expression (results not shown). No CP expression was detected from the pEAQ‐HT vector‐only negative control.

Laboratory scale plant expression studies used *Agrobacterium* LBA4404 infiltrated at OD_600_ of 1.0, with leaves harvested at 4 dpi for processing. The sucrose gradient centrifugation efficiently separated 30 mL green plant extract from a pellet containing the CP. A clear protein band at the expected PCV‐2 CP size of 27 kDa was visible when the pellet and gradient fractions were subjected to SDS‐PAGE with Coomassie Brilliant Blue staining and to immunoblotting, with soluble plant protein present in the 45% sucrose fraction 3 and CP in the pellet (Figure 1b). A 54 kDa protein was also visible in Coomassie‐stained gels with no similar proteins were present in the resuspended pellet of the pEAQ‐HT vector‐only control (Figure 1b).

Analysis of the 54 and 27 kDa protein bands using LC‐MS and comparing their profiles against a combined *N. benthamiana, A. tumefaciens, Sus scrofa* and virus proteome database, confirmed that the 27 kDa protein was purified PCV‐2 CP and indicated that the 54 kDa protein band was a PCV‐2 CP dimer (Table S1).

Independent expression experiments were compared to determine the yield of PCV‐2 CP produced in plant leaves (Figure 1c). The combined yield of the CP monomer and dimer across the independent studies ranged between 80 and 110 μg of purified PCV‐2 CP resuspended in 1 mL PBS, obtained from 15 g of leaf fresh weight homogenate in 30 mL which was loaded onto each gradient for purification (Figure 1c). This corresponds to an average of 6.5 mg of purified PCV‐2 CP obtained from 1 kg of leaf wet weight after a single sucrose gradient spin (Figure 1c).

### Density profile of plant‐produced PCV‐2 CP

**Figure 2:**
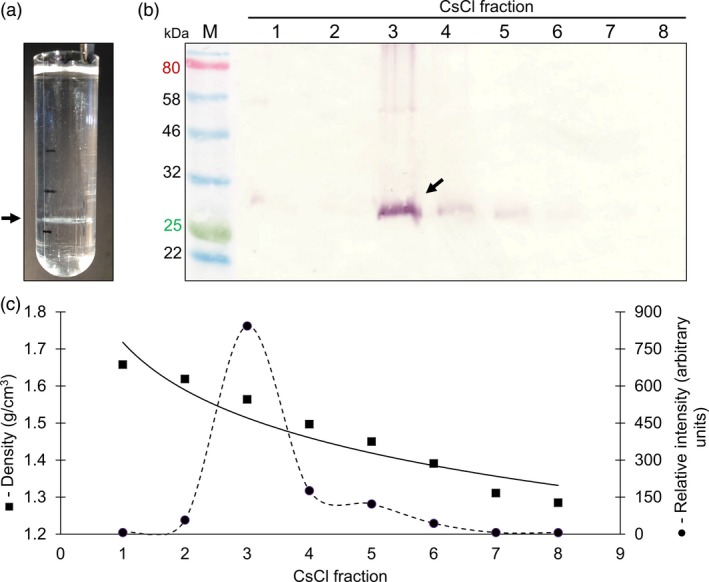
Caesium chloride density gradient profile of plant‐produced PCV‐2 CP. (a) Image of the ultracentrifuge tube after 16 h of CsCl step gradient ultracentrifugation. (b) immunoblot of collected CsCl fractions 1 to 8 from step gradient probed with rabbit anti‐PCV‐2 CP antibody. M – molecular weight marker in kilodaltons. PCV‐2 CP indicated with the black arrow. (c) the CsCl density (■) and relative intensity (●) of plant‐produced PCV‐2 CP determined from immunoblotting and densitometric analysis using the Image Studio Lite software (Version 5.2).

Resuspended pellets from the sucrose gradient were further analysed by CsCl density gradient centrifugation. After the isopycnic equilibrium was reached, a white opaque band was visible in the gradient (Figure 2a). Fractions of the density gradients ranging from 1.658–1.285 g/cm^3^ were collected for immunoblot and spectrophotometric analysis. The 27 kDa PCV‐2 CP band was detected in fraction 3 (Figure 2b) with a CsCl density of 1.564 g/cm^3^ and corresponded to the observed white band (arrow) in the CsCl gradient, with weaker PCV‐2 CP signals in fractions 4, 5 and 6 (Figure 2c).

### Confirmation of plant‐produced PCV‐2 VLPs

**Figure 3:**
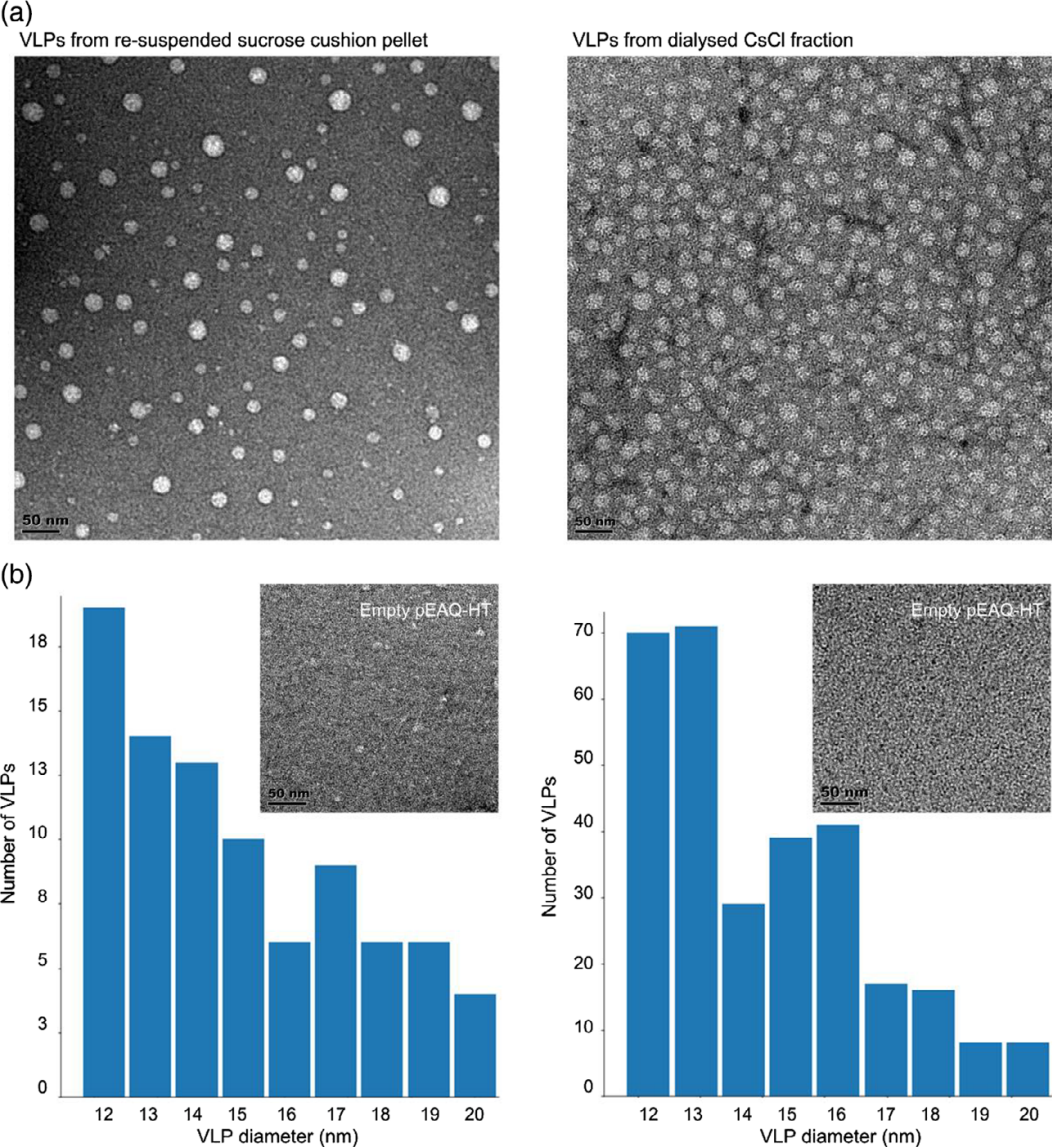
Transmission electron microscopy images and particle size distribution of plant‐produced PCV‐2 VLPs. (a) The PCV‐2 VLPs following sucrose gradient centrifugation and resuspended in PBS. Samples were loaded onto carbon‐coated copper grids and stained with uranyl acetate before viewing under TEM. The resuspended pellet sample was further analysed with CsCl centrifugation, fractions indicating PCV‐2 CP presence were dialysed overnight before viewing under TEM. (b) Corresponding histogram of size distribution (nm) of VLPs seen in the above TEM images. The image inserts are TEM images of empty pEAQ‐HT vector prepared as experimental samples and scale bar is 50 nm.

To determine whether plant‐produced PCV‐2 CP assembled to form VLPs, the resuspended pellet from the sucrose gradient as well as the CsCl‐purified fraction containing the highest CP concentration were analysed using transmission electron microscopy (TEM). In both preparations, circular particles with diameters ranging between 12 and 24 nm were observed, with the CsCl‐purified fraction having a higher concentration of particles (Figure 3a). A more thorough investigation of the size distribution of the particles based on the TEM images was performed using a proprietary image analysis particle size distribution determination package https://github.com/CorrieGunter/particle_counter. The statistical mean and median of particle sizes were 16 nm diameter, with threefold more particles detected in the image frame of the CsCl‐purified fraction (Figure 3b). No particles were present in the pEAQ‐HT vector‐only control extract.

### Immunogenicity of plant‐produced PCV‐2 CP

**Figure 4:**
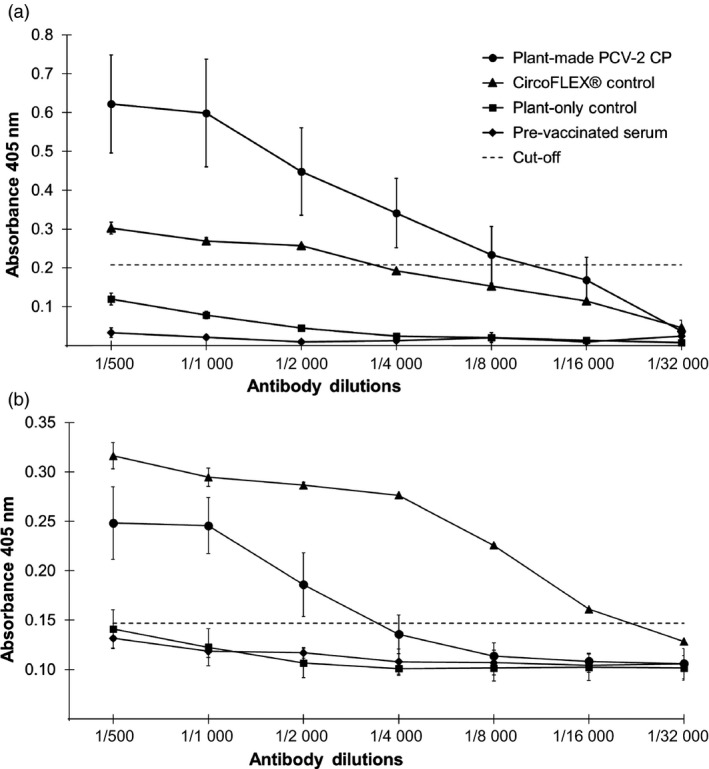
Indirect ELISA titration curve of mice serum samples. Sera, collected from the experimental and two control groups (*n* = 5), were tested in triplicate and twofold serially diluted ranging from 1 : 500 to 1 : 16 000. (a) Wells coated with plant‐produced PCV‐2 VLPs antigen. (b) Wells coated with the Ingelvac CircoFLEX ^®^ commercial vaccine antigen. The results are shown as means ± SD (*n* = 5) and cut‐off was determined from the mean OD_405 nm_ value plus 3 times standard deviation of pre‐immunized mice serum samples.

Fifteen mice were randomly divided into three groups of five and immunized with either sucrose gradient‐purified plant‐produced PCV‐2 VLPs (10–20 μg/mL), similarly fractionated pEAQ‐only infiltrated plant protein extract or Ingelvac CircoFLEX^®^ subunit‐based commercial vaccine (10–20 μg/mL). Mice immunized with the plant‐produced PCV‐2 VLPs and the commercial vaccine both showed antibodies specific for PCV‐2 CP at 42 days post immunization (Figure 4a and b). The differences in antibody titres was significantly higher compared to the plant‐only control and the pre‐immunized serum (*P < *0.01) using either plant‐produced PCV‐2 VLPs (Figure 4a) or Ingelvac CircoFLEX^®^ (Figure 4b) as antigen. No PCV‐2 specific antibodies were detected in the plant‐only immunized group 42 days after immunization or in the sera collected prior to immunization (Figure 4a and b).

## Discussion

Vaccination is the most efficient way to control, prevent and potentially eradicate PCV‐2 infections and associated diseases in swine. Insect cell, *Escherichia coli* and yeast recombinant protein production systems have shown promise for the development of viable PCV‐2 VLP‐based vaccines with the ability to elicit a strong CP‐specific immune response (Nainys *et al*., 2014; Xi *et al*., 2016). However, the expression, purification and investigation of PCV‐2 CP assembly have not previously been attempted in plants. The use of a plant expression system to produce a PCV‐2 VLP‐based candidate might also be simpler – and depending on yields, potentially more affordable – than traditionally established expression systems such as bacteria and yeast or animal cells (Sabalza *et al*., 2014).

To transiently express PCV‐2 CP in this study, the ORF2 region of the chosen genome was first codon optimized to ensure optimum tRNA use and translation by the plant cell machinery. Relative protein expression was also compared between the octopine‐catabolizing LBA4404 and succinamopine‐catabolizing EHA105 *A. tumefaciens* strains carrying the recombinant pEAQ‐HT vector. Strain EHA105 has previously been shown to have sustained, higher level recombinant protein expression to 7 dpi with fewer adverse effects in plants compared to LBA strains. However, our results showed slightly higher protein expression earlier with LBA4404 compared to EHA105 (Figure 1a) and as protein was harvested at 4 dpi, the plants agroinfiltrated with either strain showed no marked leaf pathology. The syringe‐mediated transient expression of PCV‐2 CP in plants allowed for rapid optimization of recombinant protein expression and justified progression to laboratory scale purification studies. Based on the optimization results, the pEAQ‐HT vector and *Agrobacterium* strain LBA4404 were used for further plant expression studies.

Although high‐level expression of recombinant proteins in plants is necessary to produce a competitive yield, the efficient recovery of proteins must also be considered. In our case, the 27 kDa PCV‐2 CP was concentrated in the pellet in the form of VLPs after a simple sucrose density gradient centrifugation fractionation performed on clarified plant extracts (Figure 1b). A second protein approximately double the size of the native PCV‐2 CP at 54 kDa was also seen in pellets. This suggests stable CP dimer formation, which was recently also reported for recombinant PCV‐2 CP expressed in *E. coli* (Wu *et al*., 2016) and was confirmed in this study by MS protein analysis (Table S1). This dimerization could be due to the 51–103 amino acid domain on the CP interacting during protein folding (Wu *et al*., 2012). The simple purification by sucrose gradient centrifugation led to easy detection of highly purified PCV‐2 CP in Coomassie‐stained SDS‐PAGE, which allowed for easy protein quantitation.

There was a marked difference in PCV CP yield from the same amount of plant material across two independent expression and purification studies. This variation is not uncommon for experimental plant production systems and can be attributed to plant handling conditions during purification or interactions between and storage of the expressed protein (Fahad *et al*., 2015). The highest amount of purified PCV‐2 CP obtained in this study was 6.5 mg/kg of leaf wet weight (Figure 1c). This is comparable to reported purified yields in *E. coli* of 3 mg per litre of culture (Trundova and Celer, 2007). The PCV‐2 CP was targeted to the cytosol, but total protein yield could potentially be further optimized by targeting the proteins to various cellular organelles: this has previously been demonstrated for the accumulation of plant‐produced CP for the parrot‐infecting beak and feather disease virus (BFDV; Regnard *et al*., 2017) and for human papillomavirus L1 CP (Maclean *et al*., 2007). The CsCl gradient profile obtained here showed the plant‐produced PCV‐2 CP had a buoyant density of 1.564 g/cm^3^: this differs to previous reports for PCV, which report 1.37 g/cm^3^ (Allan and Ellis, 2000; Liu *et al*., 2008). The higher density seen here could be due to denaturation and precipitation of the protein in the gradient or the possibility that the VLPs are encapsidating and packaging plant‐derived nucleic acids. Further investigation is required to ascertain the observed differences in buoyant density.

The use of VLPs to elicit neutralizing antibodies against complete virions is seen as being critical in the development of next generation PCV‐2 vaccines. It was shown elsewhere that a natural immunodominant decoy epitope on the PCV‐2 CP subunits presents to the host immune system during CP expression and subsequent protein folding in the animal host cells, allowing the virus to partially evade the host's immune response during virion formation (Trible *et al*., 2012). Mature purified PCV‐2 VLPs will have this decoy epitope embedded in the particle, however, and will display a ‘finished’ virion structure so that specific protective humoural and cellular immune responses will be generated in the host without any decoy effect (Yu *et al*., 2016). Viral CPs self‐assembling into VLPs also have the added potential to be used as display vectors to carry multimeric epitopes against different strains of the same virus (Crisci *et al*., 2012).

Transmission electron microscopy analysis of CP in sucrose and CsCl gradient‐purified fractions indicated the presence of VLPs with diameters ranging between 12 and 25 nm: these values and particle appearance match the size and morphology of PCV‐2 VLPs previously purified using *E. coli*, insect and yeast expression systems (Bucarey *et al*., 2009; Liu *et al*., 2015; Wu *et al*., 2012). The plant‐produced PCV‐2 VLPs could be effectively purified in one step from clarified plant extract by means of centrifugally pelleting them through a sucrose gradient. This purification method could be scaled up to produce antigen for serological diagnostic assays, as well as potentially for PCV‐2 vaccine manufacturing. Only a modest increase in recovery of VLPs was achieved after CsCl density gradient centrifugation, indicating the relative success of the one‐step sucrose gradient purification (Figure 1). A proportion of PCV‐2 VLPs observed by TEM was irregular in shape and size, which may be due to protein misfolding or as the result of the absence of the virus ssDNA genome. A recent study on BFDV VLPs demonstrated that in the presence of ssDNA there was stable folding of the full 12‐pentamer virion particle; in the absence of ssDNA, however, the formation of a smaller 10‐pentamer particle of 10 nm diameter was favoured (Sarker *et al*., 2016).

The PCV‐2 CP is the major candidate for vaccine development as it contains type‐specific epitopes to which neutralizing monoclonal antibodies and neutralizing swine serum antibodies have been shown to bind (Lekcharoensuk *et al*., 2004; Pogranichnyy *et al*., 2000). Recombinant VLPs which present viral antigens in a near‐authentic conformation make for better vaccine candidates and therefore the development of cost effective and safe VLP‐based vaccines for protecting swine herds is seen as being desirable. This study demonstrated that PCV‐2 CP can be produced in plants via agroinfiltration; that VLPs formed from the CP can be purified by a single step sucrose density gradient step and that these were highly immunogenic, eliciting strong PCV‐2‐specific serum immune responses in mice (Figure 4). This is the first report describing the expression and purification of PCV‐2 CP in plants, its assembly into VLPs and their immunogenicity in mice. These results indicate the significant potential of a plant expression system for the development of the next generation of PCV vaccines, as well as for manufacture of serological diagnostic reagents against currently circulating PCVs.

In summary, the high economic burden of PCV‐2 and its related diseases on the global swine industry and especially in the developing world could be reduced with the refinement of the current PCV‐2 vaccines and the investigation of novel vaccine production processes. We believe that, in this study, we have adequately demonstrated the potential of transient expression of PCV‐2 CP in plants as an exciting alternative to currently used technologies.

## Experimental procedures

### Plasmid construction

A synthesized PCV‐2 ORF2 (CP gene) derived from a Lithuanian isolate (GenBank accession number KJ128269) was plant codon optimized, the GC content was increased and the unwanted restriction enzyme sites and potential *cis*‐acting elements were removed. The restriction endonuclease sites *Age*I and *Xho*I were added on the 5′ and 3′ end of the sequence respectively for downstream cloning and the gene was provided in the pUC57 cloning vector (GenScript, Nanjing, China). The PCV‐2 ORF2 gene and pEAQ‐HT vector were independently digested with *Age*I and *Xho*I, gel resolved, purified and ligated to form the PCV‐2_pEAQ construct. Purified construct DNA was transformed into *E. coli* and selected for using 30 μg/mL kanamycin LB agar plates and incubated at 37 °C overnight. Recombinant clones were identified using colony PCR with primers: pEAQ‐HT forward 5′‐TTCTTCTTCTTGCTGCTTGG‐3′ and reverse 5′‐CACAGAAAACCGCTCACC‐3′ primer pair. The nucleotide sequence was confirmed by sequence analysis (Macrogen, Amsterdam, Netherlands) before transformation into *A. tumefaciens*.

### *A. tumefaciens* transformation

The *A. tumefaciens* strain LBA4404 was obtained from the Biopharming Research Unit culture collection (MCB, UCT) and the EHA105 strain was provided by Dr Tomáš Moravec (UEB, Czech Republic). Both *A. tumefaciens* cultures were prepared for electroporation as previously described (Shen and Forde, 1989). A 100 μL aliquot of competent cells was mixed with 400 ng of recombinant or empty (negative control) pEAQ‐HT DNA in a 1 mm gap electroporation cuvette (Bio‐Rad). The cells were electroporated (200 Ω, 1.8 kV, 25 μF) using a GenePulser (Bio‐Rad), allowed to recover at 27 °C in 1 mL LB broth for 2 h before the cells were plated on LB agar with 30 μg/mL kanamycin and 50 μg/mL rifampicin selection antibiotics and grown for 2 days at 27 °C. Transformants were screened with PCR using the pEAQ‐HT forward and reverse primers as described above and positive recombinant clones were inoculated into LB broth containing the relevant antibiotics. The empty pEAQ‐HT vector DNA served as a negative PCR control and *E. coli* derived recombinant pEAQ‐HT served as a positive control. All the cultures were supplemented with 2 mm MgSO_4_ to prevent cell clumping.

### Agroinfiltration of *N. benthamiana* leaves

Recombinant *A. tumefaciens* cultures were diluted 1 : 10 into LBB enriched medium (0.25% tryptone, 1.25% yeast extract, 0.50% NaCl, 10 mm 4‐morpholineethanesulfuric acid, pH 5.6) and incubated overnight at 27 °C with agitation. The cultures were diluted to the desired final OD_600_ in resuspension solution (5 mm 4‐morpholineethanesulfuric acid, 20 mm MgCl_2_, 0.2 mm acetosyrigone, pH 5.6) and left to stand for at least 1 h to allow the induction of Ti plasmid virulence genes. For laboratory scale experiments, anywhere between 30 and 60 plants were infiltrated from 500 to 1000 mL agrobacterium cultures. The leaves of 6‐ to 8‐week‐old *N. benthamiana* plants were vacuum infiltrated by submerging the plants into the bacterial suspension and applying a vacuum of −90 kPa before rapidly releasing the vacuum. The plants were grown under 16‐h light and 8‐h dark cycles at 22 °C.

### Preparation of plant crude extracts

The optimum *A. tumefaciens* OD_600_ and harvest day post infiltration trials were performed on a small scale and independently repeated twice. Three plants per construct and *A. tumefaciens* OD_600_ of either 0.25, 0.50 or 1.00 were vacuum infiltrated. Three leaf discs (one from each plant) were harvested, using the lid of a 1.5 mL microcentrifuge tube, on 1, 3, 5 and 7 dpi. The leaf discs were homogenized using a micropestle in the microcentrifuge tube to which 200 μL extraction DB150 buffer (150 mm NaCl, 1 mm CaCl_2_, 0.001% Triton X‐100, 0.25 mm L‐Arginine, 10% glycerol (v/v), 10 mm Tris/HCl, pH6.5) was added. The DB150 buffer was supplemented with EDTA‐free Complete protease inhibitor (Roche, Basel, Switzerland) and the samples were clarified by centrifugation at 1000 ***g*** for 5 min using a benchtop microcentrifuge. The recovered supernatant was boiled for 5 min with sample application buffer (SAB) and frozen at −20 °C until further use.

For laboratory scale extractions, the leaves of vacuum infiltrated plants were harvested, combined and weighed. Two volumes of DB150 buffer with protease inhibitor were added to the plant material before homogenizing with a T25 digital ULTRA‐TURRAX^®^ homogeniser (Sigma‐Aldrich^®^, St. Louis, MO, USA). The plant extracts were clarified by centrifugation at 8000 ***g*** for 20 min using a JA‐14 (Beckman Coulter Inc., Brea, CA) rotor and Avanti^®^ J25TI centrifuge (Beckman).

### Density gradient centrifugation purification

Both experimental plants and plants infiltrated with *Agrobacterium* containing pEAQ vector with no insert were used for protein purification. After clarifying the plant lysate by centrifugation, the supernatants were filtered through four layers of Miracloth^®^ (Merck, Kenilworth, NJ, USA). A sucrose gradient was prepared by underlaying dilutions of 6 mL (45%) and 2 mL (65%) in Thinwall 38 mL Ulta‐Clear™ ultracentrifuge tubes (Beckman). Sucrose solutions were prepared by weighing out percentage sucrose desired (for % weight/volume) and adding DB150 buffer up to the required volume. The 30 mL clarified plant extract supernatant (obtained from 15 g of leaf wet weight) was loaded onto the gradient and centrifuged using a Beckman SW 32 Ti rotor at 120 000 ***g*** and 4 °C for 4 h. The resulting liquid column was fractionated into 2 mL fractions and the pellet was resuspended in 1 mL PBS.

A sample of each fraction and the resuspended pellet was boiled for 5 min with SAB (2% SDS, 100 mm Tris‐HCl, pH 7.5, 2 mm EDTA, 52% glycerol, 4.3% β‐mercaptoethanol, 0.25% bromophenol blue), the samples were analysed with SDS‐PAGE followed by Coomassie Brilliant Blue staining or immunoblotting.

For further purification of PCV‐2 CP‐containing fractions, 4.5, 5, 5.5 and 10 g of CsCl were each dissolved in 10 mL of DB150 buffer without arginine as described by Nainys *et al*. (2014). A step gradient was prepared by underlaying the four dilutions in Thinwall 5.5 mL Ulta‐Clear™ ultracentrifuge tubes (Beckman). The resuspended pellets from the sucrose gradient were loaded onto the gradient, centrifuged in a Beckman SW 55 Ti rotor at 180 000 ***g*** for 16 h at 4 °C and fractions of 500 μL were collected as described above. The densities of CsCl fractions were confirmed using a R5000 hand refractometer (Atago, Japan).

### SDS‐PAGE, immunoblotting, protein analysis and quantification

Samples were resuspended in an equal volume of 5 × SAB and heated for 5 min at 95 °C, equal volume of each sample was always loaded in each well. The proteins were separated on 12% SDS‐PAGE at 120 V using a Tetra Cell system (Bio‐Rad, Hercules CA, USA) and were either stained for 1 h at 37 °C with Coomassie Brilliant Blue R‐250 (Sigma‐Aldrich^®^) or transferred for immunoblotting. Protein sizes were estimated with the Colour Prestained Protein Standard, broad range ladder (11–245 kDa; NEB, Ipswich, MA, USA). The separated proteins were transferred with a TransBlot^®^ Semi‐Dry Transfer Cell (Bio‐Rad) onto nitrocellulose membrane at 15 V for 1 h and treated with blocking buffer (PBS pH 7.4, 0.1% Tween‐20, 3% skim milk powder) for 30 min. Membranes were incubated with 1 : 1000 dilution of rabbit anti‐PCV‐2 serum to probe the PCV‐2 CP on immunoblots overnight at 4 °C, washed 3 × 10 min with blocking buffer and incubated with 1 : 5000 anti‐rabbit secondary antibody conjugated to alkaline phosphatase (Sigma‐Aldrich^®^). After 3 × 10 min washes in blocking buffer without skim milk, blots were developed for 30 min with NBT/BCIP substrate (Roche).

Protein identities were independently analysed by the Centre for Proteomic and Genomic Research (CPGR, South Africa). Protein bands were excised from the Coomassie‐stained SDS‐PAGE gel and fragmented by trypsin digestion alongside a BSA standard reference. The peptide solution was separated using the Dionex Ultimate 3000 Nano‐HPLC system (Thermo Fischer Scientific, MA) and then analysed using a Q Exactive™ Hybrid Quadrupole‐Orbitrap Mass Spectrometer (Thermo Fischer Scientific, Chelmsford, MA , USA). The LC‐MS generated spectra were analysed with Byonic Software (Protein Metrics, Cupertino, CA, USA) using known sequences retrieved from UniProt (www.uniprot.org). Samples were cross‐examined against a merged *N. benthamiana*,* A. tumefaciens, S. scrofa* and virus proteome databases.

Quantitation experiments used 15 g of plant leaves from independent experiments, infiltrated at *Agrobacterium* OD_600_ of 1.0, harvested at 4 dpi and resuspended in 30 mL of DB150 buffer. The plant leaves were homogenized, clarified and collected as before. The 30 mL of collected plant lysate was subjected to sucrose gradient and the collected pellets were fully resuspended in 1 mL of endotoxin‐free PBS (Sigma‐Aldrich^®^). The proteins were quantified on a Coomassie‐stained SDS‐PAGE using a twofold diluted bovine serum albumin (BSA; Sigma‐Aldrich^®^) standard curve. Protein samples, resuspended pellet and dilution series of BSA standard, were prepared by mixing and 100 μL of protein sample and 25 μL of 5 × SAB and thereafter boiled for 5 min. Equal amounts of these samples were loaded onto SDS‐PAGE for analysis. The Image Studio™ Lite version 5.2 software (LI‐COR^®^) was used to quantify the amount of protein in bands compared to the BSA standard (Figures S1 and S2).

### Transmission electron microscopy and particle size distribution determination

Glow discharged carbon‐coated copper grids (mesh size 200) were floated on samples of the sucrose purified pellets and CsCl fractions containing the plant‐produced PCV‐2 CP for 3 min at room temperature. The grids were then washed with sterile water three times, dried with filter paper and negatively stained with 2% uranyl acetate. The grids were completely dried before being viewed at 27 000–50 000× magnification with a FEI Tecnai F20 transmission electron microscope at an accelerating voltage of 120 kV. To remove the CsCl, these fractions were first dialysed in 1.5 mL microcentrifuge tubes and the caps were hollowed out and used to secure a 10 000 MW dialysis tubing (Thermo Fischer Scientific). The samples were dialysed in sterile PBS overnight at 4 °C with stirring and then loaded onto grids.

A script was compiled for obtaining a rough distribution of VLP radii from electrographs; this analysis provides a distribution of a sample of particles from the image frame (https://github.com/CorrieGunter/particle_counter). It is assumed that the VLPs are roughly circular in shape and that the TEM image contrast is reasonably consistent across the image. Python 3.5 with numpy and matplotlib including the opencv3 packages are necessary to run this script.

### Immunogenicity of PCV‐2 VLPs in mice

For the immunogenicity study and mice inoculations, plant‐produced sucrose gradient‐purified PCV‐2 VLPs and similarly fractionated empty vector control extracts were resuspended in endotoxin‐free PBS. Each dose contained approximately 10–20 μg VLPs mixed with mineral oil MONTANIDE™ ISA 50V2 in a 1 : 1 ratio. Fifteen mice were randomly divided into three groups of five and were subcutaneously injected. The first group was immunized with plant‐made PCV‐2 VLPs plus adjuvant; the second with fractionated plant protein plus adjuvant and the third group was vaccinated with 100 μL of the subunit‐based Ingelvac CircoFLEX^®^ (Boehinger Ingelheim, Germany) containing approximately the same amount (10–20 ug) of CP as the plant‐produced PCV‐2 CP (Figure S2). Each group was boosted twice at 2‐week intervals such that each group received three doses before the final serum was collected 42 days after initial immunization. All protocols were approved by the University of Cape Town Animal Ethics Committee (Application number: AEC 017‐011).

After collecting whole blood, it was allowed to clot undisturbed at 4 °C and the clot removed by centrifugation at 2000 ***g*** for 10 min, the resulting sera was used for antibody diagnostics. The pre‐immune and final serum samples were stored at −20 °C before use in ELISA experiments.

### Enzyme‐linked immunosorbent assays and cut‐off determination

The plant‐produced, gradient‐purified PCV‐2 VLPs or commercial Ingelvac CircoFLEX^®^ was used as antigen for the detection of anti‐PCV‐2 CP antibodies in mouse serum. Each well of a 96‐well plate (Thermo Fischer Scientific) was coated with either antigen overnight at 4 °C and blocked for 1 h with blocking buffer (5% skim milk and 1 × Tris buffered saline) at 37 °C. The wells were washed four times with wash buffer (0.05% Tween and 1% Tris buffered saline) before serially diluted serum samples ranging from 1 : 500 to 1 : 16 000 were incubated for 1 h at 37 °C. Wells were washed and incubated with 1 : 5000 anti‐mouse secondary antibody conjugated to alkaline phosphatase (Sigma‐Aldrich^®^). A final round of washing followed and the wells were incubated with SIGMAFAST™ p‐Nitrophenyl phosphate (Sigma‐Aldrich^®^) for 1 h in the dark. The chromogenic substrate produced a yellow colour which was measured at optical density 405 nm using a microplate spectrophotometer (PowerWave™ XS, BIO‐TEK^®^ instruments, VT). Blank, negative and positive controls were always included in each assay. The sera were analysed three times over and each sample was analysed in duplicate.

The cut‐off was determined from the mean OD_405 nm_ value plus three times standard deviation of pre‐immunized mice serum samples for each ELISA. Statistical significance was compared between all experimental groups with the Excel Microsoft Office 365 ProPlus software using the Data Analysis package.

## Conflicts of interest

The authors declare no conflicts of interest.

## Supporting information


**Figure S1** Quantification of plant‐produced PCV‐2 CP (27 kDa).

**Figure S2** Quantification of Ingelvac CircoFLEX^®^ commercial vaccine and plant‐produced PCV‐2 CP (27 kDa).

**Table S1** Protein identities from 27 and 54 kDa SDS‐PAGE gel band analyses.
