# Supplementary material for: Immunogenicity of plant‐produced porcine circovirus‐like particles in mice
Source: Plant Biotechnol J. 2019 Mar 10;17(9):1751–9. doi: 10.1111/pbi.13097 (PMC6686138; doi:10.1111/pbi.13097)
Supplement: Supplementary file 1 — Figure S1 Quantification of plant‐produced PCV‐2 CP (27 kDa). [file PBI-17-1751-s001.pdf]

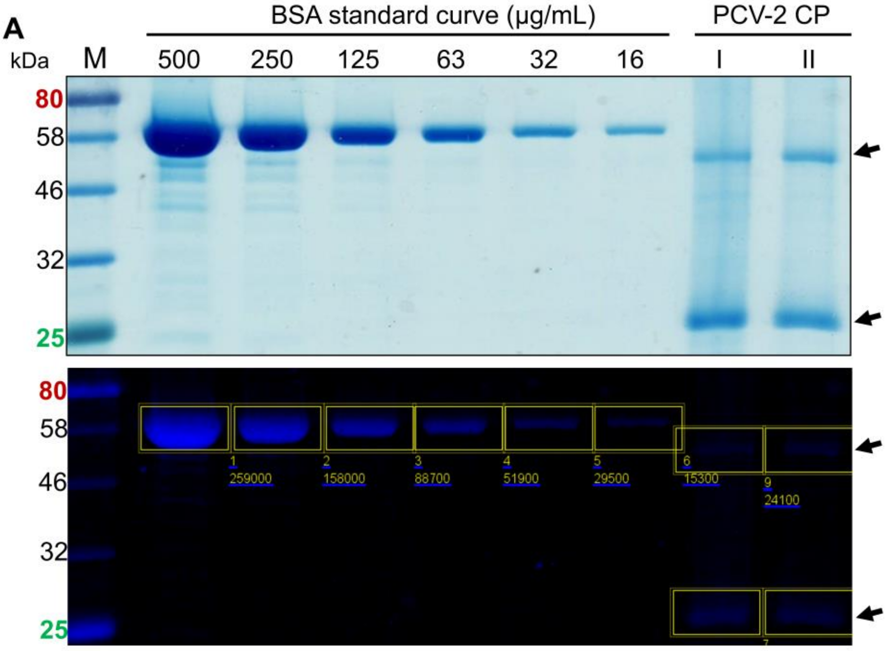

B

BSA standard (µg/mL)	Signal
500	247756
250	148072
125	82644
63	51006
31	24177
16	15208
**Sample I**	**70428**
**Sample II**	**55350**

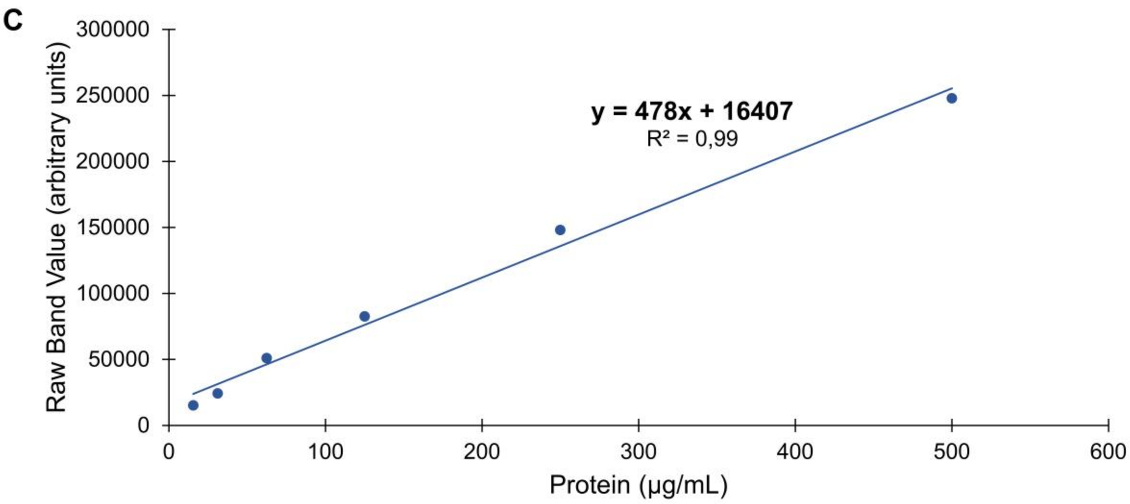

Figure S1: Quantification of plant-produced PCV-2 CP (27 kDa).

A - Two independently expressed and partially purified recombinant 27 kDa PCV-2 CP samples I and II resolved on Coomassie Blue stained SDS-PAGE for densitometric analysis with Image Studio Lite software (Version 5.2). Equal volume of BSA protein standard and samples were loaded per well. Amount of BSA (µg/mL) per well is indicated above each lane. B – Raw band value of independently produced PCV-2 CP samples for protein quantification. C – Densitometry analysis with R^2 value and equation used for quantification of recombinant PCV-2 CP. Molecular weight marker (M).
