# Supplementary material for: Immunogenicity of plant‐produced porcine circovirus‐like particles in mice
Source: Plant Biotechnol J. 2019 Mar 10;17(9):1751–9. doi: 10.1111/pbi.13097 (PMC6686138; doi:10.1111/pbi.13097)
Supplement: Supplementary file 2 — Figure S2 Quantification of Ingelvac CircoFLEX® commercial vaccine and plant‐produced PCV‐2 CP (27 kDa). [file PBI-17-1751-s002.pdf]

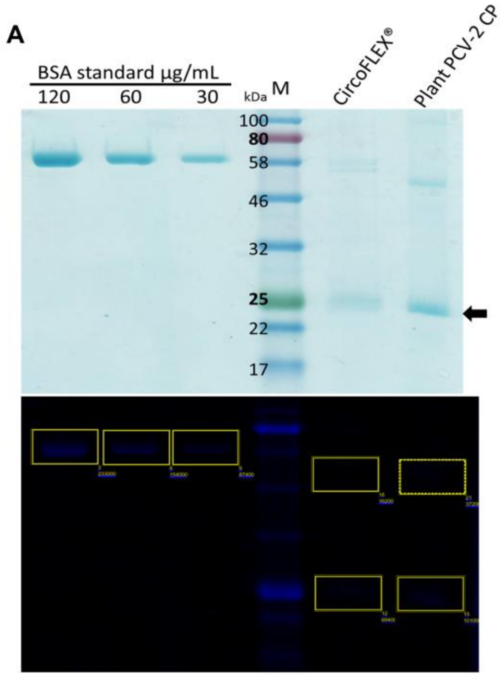

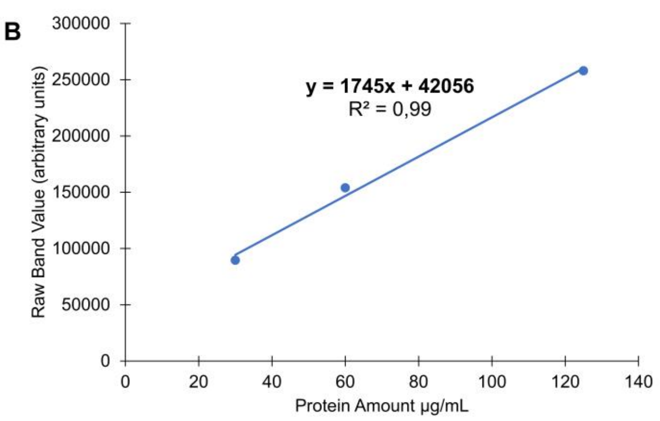

C

Lane		Signal	
BSA (µg/mL)	125	257899	
	60	153920	
	30	89495	**µg/mL**
CircoFLEX®		73486	**18**
Undiluted plant-made PCV-2 CP		101900	**34**

Figure S2: Quantification of Ingelvac CircoFLEX® commercial vaccine and plant-produced PCV-2 CP (27 kDa).

A - CircoFLEX® commercial subunit-based vaccine and plant-produced partially purified on a sucrose gradient recombinant PCV-2 CP sample was resolved on Coomassie Blue-stained SDS-PAGE with BSA protein standard for densitometric analysis with Image Studio Lite software (Version 5.2). B – Densitometry analysis with R^2 value and equation used for quantification of Ingelvac CircoFLEX® and plant-produced PCV-2 CP. C – Table with raw band value of Ingelvac CircoFLEX® and plant -produced PCV-2 CP samples for protein quantification. Molecular weight marker (M).
