# Supplementary material for: Immunogenicity of plant‐produced porcine circovirus‐like particles in mice
Source: Plant Biotechnol J. 2019 Mar 10;17(9):1751–9. doi: 10.1111/pbi.13097 (PMC6686138; doi:10.1111/pbi.13097)
Supplement: Supplementary file 3 — Table S1 Protein identities from 27 and 54 kDa SDS‐PAGE gel band analyses. [file PBI-17-1751-s003.pdf]

Protein Rank	Description	Best score	Unique peptides	AA's in protein	Protein DB number
**27 kDa protein band**					
1	PCV2 Capsid protein OS=Porcine circovirus 2	727,80	22	233	26158
2	PIG Uncharacterized protein OS=*S. scrofa*	481,20	6	573	10063
3	PIG Trypsin OS=*S. scrofa*	631,90	4	231	1364
3	PIG Uncharacterized protein OS=*S. scrofa*	631,90	4	231	1364
4	PIG Uncharacterized protein OS=*S. scrofa*	384,00	3	580	8503
6	PIG Uncharacterized protein OS=*S. scrofa*	383,20	2	629	11836
**54 kDa protein band**					
1	PCV2 Capsid protein OS=Porcine circovirus 2	545,20	16	233	26158
2	PIG Trypsin OS=*S. scrofa*	409,90	4	231	1364
2	PIG Uncharacterized protein OS=*S. scrofa*	409,90	4	231	1364
3	PIG Uncharacterized protein OS=*S. scrofa*	344,80	5	573	10063
4	PIG Uncharacterized protein OS=*S. scrofa*	507,20	3	610	17983
5	PIG Uncharacterized protein OS=*S. scrofa*	307,30	3	475	11229
6	PIG ATP synthase subunit alpha, mitochondrial OS=*S. scrofa*	186,10	2	553	145

Table S1: Protein identities from 27 and 54 kDa SDS-PAGE gel band analyses.

Table shows top six returned identities of the 27 and 54 kDa bands excised from SDS-PAGE gel and fragmented by trypsin digestion. The peptide solution was separated using HPLC system and analysed using a mass spectrometer. The samples were cross-examined against a merged *N. benthamiana, A. tumefaciens, S. scrofa* and virus proteome database. Protein description with species origin (OS), best score, number of unique peptides, number of amino acids in protein and the UniProt protein data base numbers are given. The PIG trypsin from *S. scrofa* is likely an artefact from the trypsin digestion.
